# Role of Direct vs. Indirect Pathways from the Motor Cortex to Spinal Motoneurons in the Control of Hand Dexterity

**DOI:** 10.3389/fneur.2013.00191

**Published:** 2013-11-19

**Authors:** Tadashi Isa, Masaharu Kinoshita, Yukio Nishimura

**Affiliations:** ^1^Department of Developmental Physiology, National Institute for Physiological Sciences, Okazaki, Japan; ^2^Department of Life Sciences, Graduate University for Advanced Studies (SOKENDAI), Hayama, Japan; ^3^Precursory Research for Embryonic Science and Technology, Japan Science and Technology Agency, Saitama, Japan

**Keywords:** corticospinal tract, propriospinal neuron, precision grip, macaque monkey

## Abstract

Evolutionally, development of the direct connection from the motor cortex to spinal motoneurons [corticomotoneuronal (CM) pathway] parallels the ability of hand dexterity. Damage to the corticofugal fibers in higher primates resulted in deficit of fractionated digit movements. Based on such observations, it was generally believed that the CM pathway plays a critical role in the control of hand dexterity. On the other hand, a number of “phylogenetically older” indirect pathways from the motor cortex to motoneurons still exist in primates. The indirect pathways are mediated by intercalated neurons such as segmental interneurons (sINs), propriospinal neurons (PNs) reticulospinal neurons (RSNs), or rubrospinal neurons (RuSNs). However, their contribution to hand dexterity remains elusive. Lesion of the brainstem pyramid sparing the transmission through the RuSNs and RSNs, resulted in permanent deficit of fractionated digit movements in macaque monkeys. On the other hand, in our recent study, after lesion of the dorsolateral funiculus (DLF) at the C5 segment, which removed the lateral corticospinal tract (l-CST) including the CM pathway and the transmission through sINs and RuSNs but spared the processing through the PNs and RSNs, fractionated digit movements recovered within several weeks. These results suggest that the PNs can be involved in the recovery of fractionated digit movements, but the RSNs and RuSNs have less capacity in this regard. However, on closer inspection, it was found that the activation pattern of hand and arm muscles considerably changed after the C5 lesion, suggesting limitation of PNs for the compensation of hand dexterity. Altogether, it is suggested that PNs, RSNs RuSNs, and the CM pathway (plus sINs) make a different contribution to the hand dexterity and appearance of motor deficit of the hand dexterity caused by damage to the corticofugal fibers and potential of recovery varies depending on the rostrocaudal level of the lesion.

## Introduction

A generally accepted concept on the neuronal mechanism of hand dexterity is that the direct corticomotoneuronal (CM) connection, which first appears in the higher primates during evolution, plays a major role in the control of hand dexterity such as the potential to control fractionated digit movements as represented by precision grip (1–4). This concept is primarily derived from (1) parallel development of the fractionated digit movements and the CM pathway (5–8), and (2) observation of human patients and lesion studies in non-human primates (9–12); stroke that affects the corticofugal fibers severely affects the fractionated digit movements in patients, and lesion of the pyramidal tract at the brainstem level permanently impairs the hand dexterity in monkeys. In addition, some of the CM cells were shown to be specifically activated during the precision grip, but not during the power grip, even though their target muscles were similarly activated (13).

On the other hand, the corticofugal fibers issue collaterals to targets at various rostrocaudal levels besides spinal motoneurons (14, 15). Thus, the direct CM pathway is not the exclusive route to spinal motoneurons, instead there are several indirect routes, which could mediate the cortical command to hand and arm motoneurons located in the lower cervical segments (C6-Th1). First, the neurons in the magnocellular red nucleus (16, 17) and the ponto-medullary reticular formation (18–21) mediate the cortical command to motoneurons via the rubrospinal tract (RuST) and reticulospinal tract (RST), respectively. In addition, it was recently determined that the propriospinal neurons (PNs) in the mid-cervical segments and segmental interneurons (sINs) in the cervical enlargement (C6-Th1) could relay the cortical inputs to motoneurons (22, 23). Moreover, the existence of the pathway from the ipsilateral cortex to spinal motoneurons, mediated by reticulospinal neurons (RSNs), has been shown in cats (24). To date, these indirect pathways have been considered not to contribute to the control of fractionated digit movements because a major advantage of the direct CM pathway was actually to bypass the indirect routes, which were considered to have too widespread connections with distal motoneurons to mediate the command for independent digit movements (25). However, recently, it has been clarified that the PNs are involved in the control of dexterous digit movements in the normal state (26, 27) and also in the recovery of precision grip after lesion of the direct CM connection. In this manuscript, the current views on the role of these indirect pathways in the control of hand dexterity in the normal state and during functional recovery after damage to the corticospinal tract will be discussed.

## Individual Indirect Routes; Anatomy and Physiology

**Figure 1:**
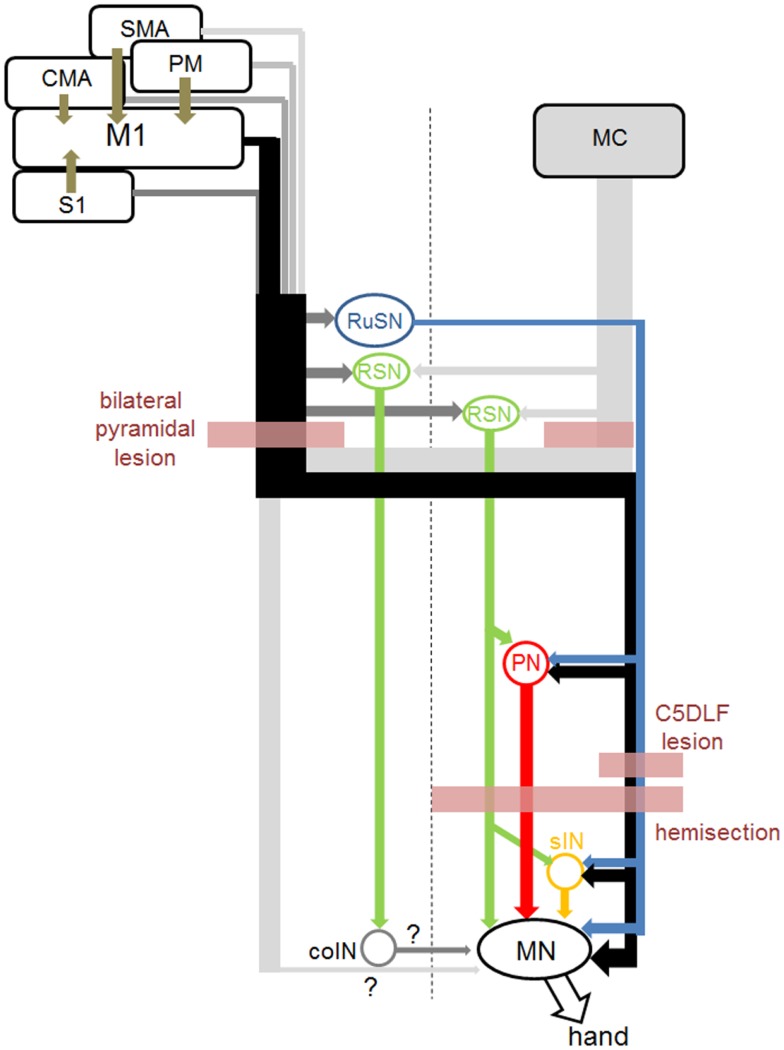
**Direct and indirect pathways from the sensorimotor cortices to hand motoneurons, and sites of lesion in the studies cited in this review**. Connection from the midline-crossing axons from the contralateral corticospinal tract and commissural interneurons (coINs) was not clarified before (marked as “?” in this figure). Connection from the RuSNs to the PNs and sINs, and that from the RSNs to PNs were demonstrated in cats, but not in primates yet.

In this section, first, we will review the anatomy and physiology of several indirect routes from the motor cortex to spinal motoneurons (Figure 1). The direct CM pathway is primarily originated from the primary motor cortex (M1), especially in the region along the bank of the central sulcus [new M1; (28)]. On the other hand, the whole M1 (both the bank and convexity regions) and other sensorimotor cortices such as premotor (PM), supplementary (SMA), cingulate (CMA), primary somatosensory (S1) areas send descending axons to the subcortical centers and intermediate zone of the spinal cord and might contribute to the indirect pathways (15, 29–33).

### Cortico-rubrospinal pathway

The RuST originates from the magnocellular subdivision of the red nucleus in the ventral midbrain. The magnocellular red nucleus markedly develops in the reptile, birds, and lower mammals, but becomes less evident in higher primates including humans (34). In the cat, the direct connection of the RuST with spinal motoneurons is limited to those innervating the most distal muscles (35) but the rubrospinal neurons (RuSNs) have been shown to be connected mainly to spinal interneurons and modulate the spinal reflexes (36). In primates, the RuST makes direct connection with motoneurons of wrist muscles and control dynamic phase of movements (16). Cortical input to the magnocellular red nucleus is mainly originated from the M1 (17).

### Cortico-reticulospinal pathway

The RST is originated from the medial ponto-medullary reticular formation and is a phylogenetically old descending motor pathway. It has long been considered to control the movements of proximal muscles such as head, trunk, and proximal limbs rather than distal muscles in cats (37, 38). However, recent studies have shown that these pathways are also involved in the control of hand movements in primates. Davidson and Buford studied the stimulus triggered and spike-triggered averaging of EMG activity following activation of the medial ponto-medullary reticular neurons in awake monkeys and showed short latency excitation in shoulder and arm muscles (18, 19). Baker and colleagues showed that electrical stimulation of the medial ponto-medullary reticular formation evoked mono- or oligosynaptic excitation in motoneurons innervating distal muscles and spinal interneurons in the lower cervical segments in macaque monkeys (20, 39). They also showed a group of reticular neurons that exhibited activity closely related to slow finger movements (40). Cortical inputs to these reticular neurons have been studied by neuroanatomical techniques (29, 41). These results suggested the possible contribution of RSNs in primates to the control of hand movements (42).

### Cortico-propriospinal pathway

The propriospinal relay of cortical commands for the control of hand and arm movements has been extensively studied by Lundberg and colleagues in cats, which lack the direct CM connection (43–46). Electrical stimulation of the brainstem pyramid induced disynaptic excitation in forelimb motoneurons in anesthetized cats (47). To clarify the segmental location of intercalated neurons mediating the disynaptic excitation, selective lesion of the lateral corticospinal tract (l-CST) was made by transecting the DLF at various rostrocaudal levels. It was found that substantial amount of excitation still remained after the l-CST lesion at the C5 level, whereas the excitation almost completely disappeared after the C2 lesion, which suggested the involvement of PNs in the C3-C4 segments (C3-C4 PNs) in the control of forelimb movements besides the sINs. Cell bodies of the PNs are mainly distributed in the lateral part of the laminae VI, VII, and VIII of the C3-C4 segments and their descending axons are located in the ventral part of the lateral funiculus and lateral part of the ventral funiculus. Function of the PNs and sINs was assessed by behavioral observation with the l-CST lesion at C5 or C2 segment (48). The cat with the l-CST lesion at C5 exhibited deficit in the grasping movements, whereas both reaching and grasping were impaired in those with the C2 lesion. Based on these findings, it has been proposed that PNs are mainly involved in the control of reaching, while grasping is primarily controlled by the sINs. In macaque monkeys, disynaptic pyramidal excitatory excitation via PNs was found in relatively small number of motoneurons and it was questioned whether there was significant transmission of corticospinal excitation of hand motoneurons through this route (49–51). However, it was shown that disynaptic excitation of motoneurons could be observed when glycinergic inhibition was reduced by intravenous administration of strychnine in anesthetized macaque monkeys (22) and the C3-C4 PNs which were antidromically activated from forelimb motor nuclei became orthodromically activated by the pyramidal stimulation after strychnine injection (23). These results suggested that cortical command could be mediated to motoneurons by the C3-C4 PNs, however, strong feedforward inhibition, which might be unique in primates, masked the pathway.

### Cortico-segmental interneuronal pathway

In cats, it has been shown a population of sINs mainly located in laminae VI and VII of the forelimb segments (C6-Th1) were orthodromically activated from the CST and exert postspike effects on hand motoneurons (52). In primates, Fetz and colleagues developed a technique of single unit recording from cervical spinal cord in awake monkeys performing wrist flexion-extension task. They reported a group of sINs which showed increase or decrease in activity in relation to the wrist movements (53). Moreover, some of them were shown to exhibit postspike effects on EMG activity of wrist muscle with spike-triggered averaging (54). These results suggest that a population of sINs mediate commands for wrist flexion or extension to wrist motoneurons in monkeys (55). More recently, Takei and Seki (56, 57) showed that a group of premotor sINs, identified by the postspike effects on the EMG activity of hand muscles, modulated their activities during precision grip task. These lines of studies suggest that the sINs could mediate commands for fractionated digit movements to motoneurons. In both lines of studies, presence of the cortical input to these INs was not demonstrated, which needs to be assessed in future studies.

### Descending pathways from the ipsilateral motor cortex

Contribution of the ipsilateral motor cortex to forelimb movements has been investigated in several studies [e.g., Ref. (58)]. In primates a recent study intensively investigated whether the ipsilateral motor cortex is involved in the control of forelimb movements by applying a variety of techniques and eventually concluded that there is no evidence that the ipsilateral motor cortex controls the movements (59). In normal primates, stimulation of the ipsilateral pyramid induced no effects on membrane potential of forelimb motoneurons (59). On the other hand, Jankowska and colleagues showed that stimulation of the ipsilateral brainstem pyramid induced oligosynaptic excitation in hindlimb motoneurons after administration of 4-aminopyridine to increase the excitability of the neural circuits in anesthetized cats. Subsequently they analyzed the pathway mediating the effect and showed that RSNs which descend through the contralateral spinal cord mediate the inputs from the ipsilateral brainstem pyramid to the motoneurons via the commissural interneurons (coINs) in the lumbar spinal cord (24). Thus, there might exist an oligosynaptic pathway from the ipsilateral motor cortex to forelimb motoneurons, which would be unmasked by pharmacological manipulation to enhance excitability of the pathway. On the other hand, reversible blockade of ipsilateral M1 caused no effects on precision grip movements in intact monkeys (60), however, the same manipulation was shown to impair the hand movements during early stage of recovery after C5 l-CST lesion (60), suggesting the potential of the ipsilateral motor cortex to control hand muscles during recovery from the spinal cord injury. Similar pharmacological manipulation as used by Jankowska and colleagues in cats should be tested in monkeys in future to investigate the pathway to resolve this discrepancy.

## Lesion Models of the Corticofugal Fibers in Non-Human Primates

In this section, we will review the literature describing the recovery of hand and arm movements, especially of their dexterity, following lesion of the corticofugal fibers mainly in non-human primate models (Figure 1). As shown below, the potential of recovery of the dexterous digit movements is markedly different depending on the level of the lesion, which might be due to the available indirect pathways in each case. We will also discuss on the possible neural pathways which might be responsible for the recovery. Here we have to note that location of lesions made to assess the function of individual systems was different, e.g., in some cases by lesioning the input pathway and in other cases by lesioning the output pathway.

### Brainstem pyramid

Many classical lesion experiments were performed at the level of the brainstem pyramid [“pyramidotomy”; (9–12)]. In these studies, fractionated, independent digit movements were severely impaired. The deficit in the studies by Lawrence and Kuypers (11) was generally less severe compared with other studies. But still, recovery was observed only in movements of more proximal part of the arm and less fractionated movements of the hand. The authors hypothesized that the recovered movements must be controlled by the brainstem routes which were spared by the pyramidal lesion. To test the involvement of the brainstem pathways in recovery, they made additional lesion of the lateral brainstem pathway and ventromedial brainstem pathway either at the brainstem or in the spinal cord, respectively (12), and found that interruption of the lateral brainstem pathway (presumably the RuST) resulted in impairment of distal extremity and hand movements, while interruption of the ventromedial pathways (presumably RST) resulted in a severe impairment of movements in axial muscles and proximal extremities. Based on these findings, the recovery of hand movements following the pyramidotomy was supposed to be chiefly compensated by the RuST. In humans, Bucy et al. (61) reported the results of transection of the cerebral peduncle in patients with dyskinesia and showed recovery of limb movements, which was considered partly due to the spared corticospinal fibers but mainly due to the subcortical pathways.

On the other hand, more recently, Baker and colleagues investigated the role of the RST in the control of distal hand movements and showed that after recovery from lesion of the brainstem pyramid, “the lesioned animals had recovered to a high level of gross motor function as described previously (12).” but showed “no evidence of relatively independent finger movements” (62). The authors analyzed the amplitude of the EPSPs evoked by electrical stimulation of the medial ponto-medullary reticular formation in hand muscles became facilitated, which suggested plasticity in the RST for compensation of the impairment of the corticospinal inputs to the spinal circuits. Based on these findings, it is suggested that the RST is responsible for some recovery of hand movements after stroke but only for gross motor function of hand, not for fractionated digit movements.

### Mid-cervical segment (C5)

To study the role of the PNs in the control of fractionated digit movements, we investigated the effect of l-CST lesion at the C5 segment in macaque monkeys. First, selective lesion of the DLF which would have interrupted the l-CST was made at the C5 segment. Immediately after the lesion, the monkeys showed some deficit in fractionated digit movements, which confirmed the role of the l-CST input to the lower cervical segments in the control of hand dexterity. However, some of independent finger movements could still be observed immediately after the lesion (<2 weeks) in all the animals with lesions that matched the location of the l-CST. In the monkeys with C5 l-CST lesions, including the animals with larger lesions which invaded into the more ventral aspect of the lateral funiculus, precision grip recovered in a few weeks to a few months. Until now, we have tested the similar lesion in more than 20 monkeys and all of them showed recovery of precision grip to the success ratio higher than 90%. On the other hand, as described in the previous section, pyramidal lesion at the brainstem level showed limited recovery of fractionated digit movements as reported by Lawrence and Kuypers (11). Thus, comparison of the recovery after the l-CST lesion at the C5 and brainstem suggested that the PNs located rostral to the C5 are involved in the control of fractionated digit movements and also their long term recovery after the l-CST lesion at C5.

Cortical activation during the course of recovery after the C5 lesion was investigated with positron emission tomography (PET) (60, 63). It was revealed that during the early period of recovery (1 month postoperative), activity of bilateral M1 hand areas were increased in comparison to the preoperative state, while at the late stage of the recovery (3–4 months postoperative), the activation of the contralesional M1 was enhanced and expanded, and bilateral PMv also increased the activation. Causal relationship of these areas to recovery was tested by reversible blockade of these cortical areas with microinjection of muscimol. It was found that contralesional M1 was involved in recovery during both early and late recovery stages, ipsilesional M1 only during the early stage and ipsilesional PMv only during the late stage. Thus, depending on the recovery stages, the contribution of different cortical areas to the recovery changed. How these areas are involved in the recovery, namely the pathway from these cortical areas to the hand motoneurons of the affected limb, remains unclear.

The effect of early rehabilitative training was investigated in the C5 l-CST lesion model (64). In one group of animals with the l-CST lesion, the affected forelimb was restrained for 1 month and the rehabilitative training was then initiated. The progress of recovery of hand dexterity in these animals was compared 3 months after initiation of the training with the animals in which the training was started immediately after the lesion. The recovery was much worse in the animals with late onset of training, which suggests that the early rehabilitative training is important for better recovery of motor function after the spinal cord injury.

### Lower cervical hemisection

The effects of hemisection at the lower cervical level (C5-C7) have been studied by several groups (65–69). These studies showed that initially the monkeys exhibited severe flaccid hemiparesis during the first month and remarkable recovery of grasping movements of the affected limb occurred during the following several months. However, the deficit in the hand dexterity remained. Galea and Darian-Smith (65) studied the possibility of regeneration of severed corticospinal axons, however they could not clearly show it, and suggested that the incomplete recovery must be achieved by optimization of signal transmission from the cortex to spinal cord via the reduced population of corticospinal and corticobulbospinal projection or effective use of the spinal circuitries. More recently, Rosenzweig et al. (68) showed extensive sprouting of midline-crossing axons from the corticospinal fibers descending in intact side of the spinal cord, which may underlie the recovery of hand movements (68).

In humans, Nathan and Smith (70) reported the cases of cordotomies for the treatment of pain from cancer. When the first large cordotomy was performed unilaterally to incise almost all the descending tracts on one side (or plus those in the ventral funiculus on the contralateral side), it caused severe paresis in the ipsilateral hindlimb, but the paresis regressed and limb movements showed good recovery. However, if the second cordotomy was carried out on the contralateral side, the recovery that had occurred was immediately removed and little re-recovery occurred. These results suggested the contralateral corticospinal tract, whose fibers must recross the cord should be responsible for the recovery, which is supported by the above mentioned observation in the non-human primate model.

## Role of the PNs in the Intact State

**Figure 2:**
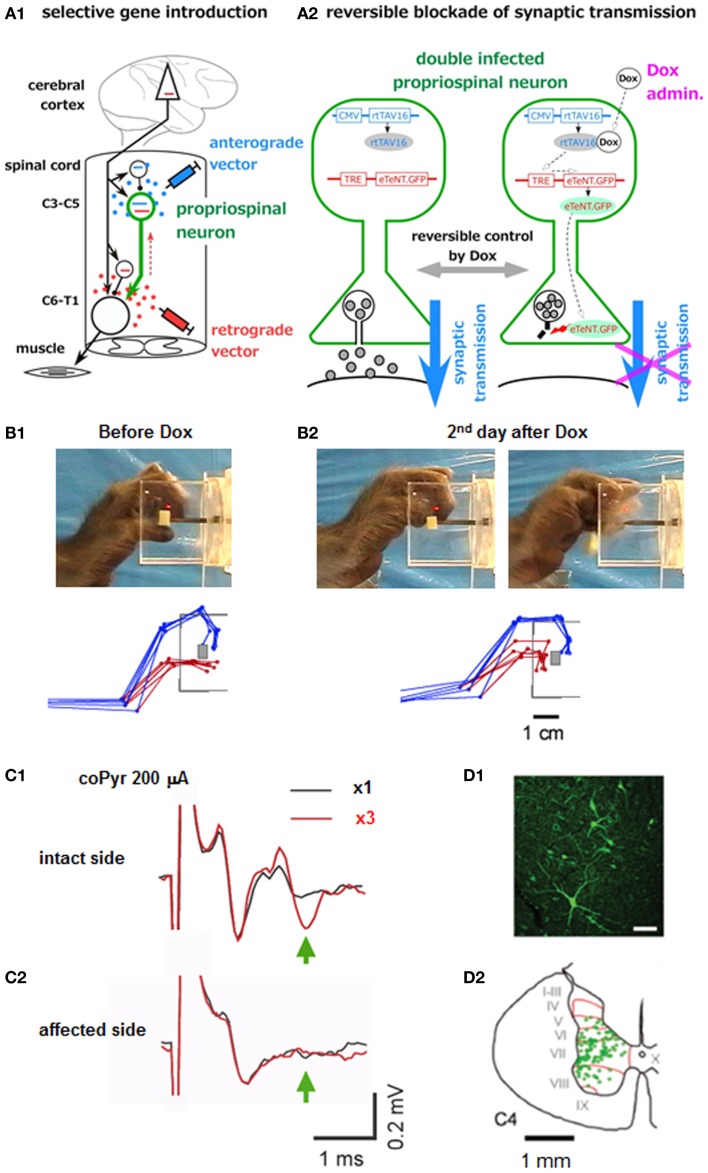
**Pathway-selective and reversible blockade of synaptic transmission using double infection with viral vectors**. **(A1)** Experimental arrangements, **(A2)** How the gene sequences carried by the two vectors interact with each other under the presence of Dox and block the synaptic transmission. **(B1)** Photo image and superimposition of stick diagrams of precision grip before Dox administration. Blue sticks indicate the index finger and red sticks indicate the thumb at the timing of grasping a small piece of sweet potato. **(B2)** The same arrangement taken on the second day after the start of Dox administration. **(C1)** Local field potential in the DR motor nucleus, evoked following the first stimulus of the brainstem pyramid (at 200 μA) (blue) and that following the third stimulus (red) applied at 300 Hz on the intact side. **(C2)** The same arrangement. On the affected side. **(D1)** Fluorescent view of the GFP-positive neurons (PNs). **(D2)** Laminar distribution of the GFP-labeled cells in the C4 segment [modified from Ref. (27)].

The recovery of precision grip after lesion to the corticospinal tract was markedly different between the lesion at C5 and brainstem; the ability of precision grip recovered after C5 lesion but not after the pyramidal tract lesion at the brainstem. These results suggested that the PNs rostral to the C5 segments, presumably the C3-C4 PNs contributed to the recovery of fractionated digit movements. However, substantial amount of plastic change should occur during the recovery process in the network organization [e.g., Ref. (60, 71–73)] and the above results do not indicate the normal function of the PNs. As described in the preceding section, trace of independent control of finger movements immediately after the lesion (<2 weeks) greatly differed between the l-CST lesion at C5 and C2 (26); it remained after the C5 lesion, while not after the C2 lesion. We suggested that the C3-C4 PNs are involved in the control of fractionated digit movements from these findings, however this was still based on indirect evidence. More direct evidence could be obtained by reversible and selective blockade of the C3-C4 PNs themselves, however dissection of such particular neuronal population, mixed with other neuronal populations locally, was not possible before. Recently we succeeded in developing a new method of pathway-selective and reversible blockade of synaptic transmission by using double infection with viral vectors and applied it to the C3-C4 PNs in macaque monkeys (27). First, the highly efficient retrograde gene transfer vector (HiRet) (74), which is a modified lenti-viral vector pseudotyped with the chimera of glycoprotein of rabies virus and vesicular stomatitis virus, carrying the tetracycline responsive element (TRE), enhanced tetanus neurotoxin (eTeNT), and enhanced green fluorescent protein (EGFP) (HiRet-TRE-EGFP.eTeNT), was injected into the ventral horn of the C6-Th1 segments, where the motor nuclei of hand and arm muscles are located. About 7–10 days later, the second vector, adeno-associated viral vector type 2 carrying cytomegalovirus promotor (CMV) and newly developed highly efficient Tet-ON sequence, rtTAV16 (AAV2-CMV-rtTAV16), into the intermediate zone of the C3-C4 segments (Figure 2A1). With this arrangement, eTeNT was expected to be specifically expressed in the PNs in the presence of doxycycline (Dox) (Figure 2A2). Then, about 1–2 months later, Dox was orally administered to the monkey for 1 week and deficit in precision grip and/or accuracy in reaching on the 2–5 days after the start of the administration; failure rate increased and/or the movement time was prolonged (Figures 2B1,2). When Dox was administered repeatedly at 1 month intervals, the deficit was observed again. On the final day of Dox administration, the monkey was anesthetized and terminal electrophysiological experiments were performed. When extracellular local field potential was recorded in the motor nuclei innervating the deep radial (DR) nerve and electrical stimulation was applied to the brainstem pyramid contralateral to the recordings, a short latency negative field potential, which is supposed to include the direct conduction volley and subsequent negative field potential reflecting the disynaptic excitation, could be observed on the intact side. The latter component is supposed to include the components mediated by the sINs, PNs, and RSNs. Therefore, we made the l-CST lesions at the C5 and subsequently at the C2 and the difference between the conditions with the C5 and C2 lesion was interpreted to indicate the component mediated by the PNs. The disynaptic excitation mediated by the PNs thus estimated was by 90% reduced on the affected side compared to the intact side (Figures 2C1,2), in contrast to the monosynaptic component which was not statistically different. In the end, because the expressed eTeNT and EGFP composed the fusion protein, the neurons which expressed the eTeNT was labeled with EGFP. The neurons labeled by anti-GFP immunohistochemistry were distributed mainly in the lateral portion of lamina VI and VII, which well fitted with the previous knowledge on the location of the premotor PNs (Figures 2D1,2). Moreover, because the eTeNT exerts its effect after transported to the nerve terminal, the whole axonal trajectories could be visualized by the anti-GFP immunohistochemistry. Thus, many axons descending to the forelimb segments (C6-Th1) and terminal buttons were found to be attached to the presumed motoneurons in the ventral horn, which confirmed that the premotor PNs were actually affected by the gene introduction. All the above findings strongly suggest that the PNs are involved in the control of dexterous digit movements in primates in the intact state. On the other hand, it should be stressed that some PNs have ascending axons toward the lateral reticular nucleus (LRN) in the medulla (23). This ascending pathway has been considered to carry the efference copy signal to the cerebellum. Actually a large number of GFP-positive buttons could be found in the LRN. Such observation suggested that the behavioral deficits are explained both by the blockade of signal transmission toward the motoneurons, and by the blockade of the efference copy signal. This remains a question to be tackled in future studies.

## Limitation of Indirect Pathways in the Control of Hand Dexterity

**Figure 3:**
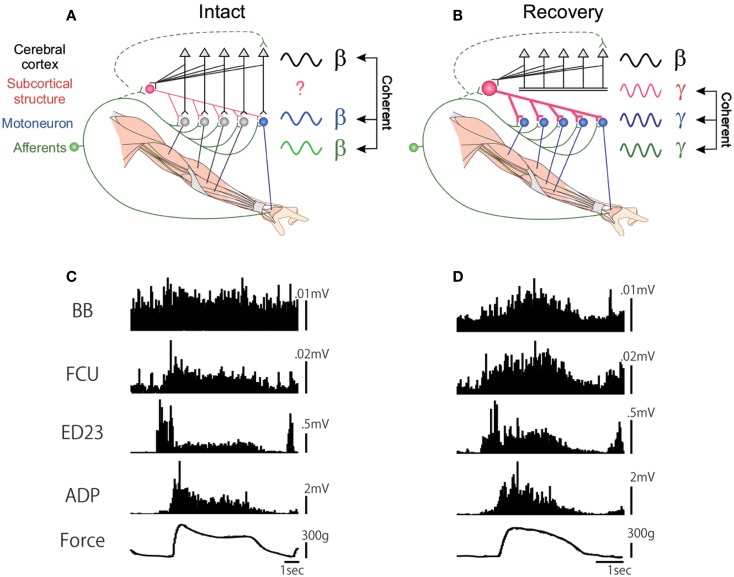
**EMG activity of proximal and distal forelimb muscles during the force-tracking precision grip task in a monkey before and 91 days after the D5 l-CST lesion**. **(A)** Schematic drawing of the cortico-muscular coherence (at β-band) in the intact state. **(B)** EMG recordings during the force-tracking precision grip task before lesion. **(C)** Lack of cortico-muscular coherence but appearance of musculo-muscular coherence (at γ-band) after recovery from the C5 l-CST lesion. **(D)** EMG recordings during the force-tracking precision grip task 91 days after lesion. FCU, flexor carpi ulnaris; ED2,3, extensor digitorum 2,3; ADP, adductor pollicis [modified from Ref. (73)].

As described above, the PNs in primates are involved in the control of fractionated digit movements. But is there any difference in their capacity to control the fractionated digit movements between the direct CM pathway and the PN-mediated indirect pathway? We recorded the EMG activity of both proximal and distal muscles in the affected hand and analyzed the pattern of muscle activation during the force-controlled precision grip task before and after the l-CST lesion at C5 (73). As shown in Figure 3, adductor pollicis (ADP) and extensor digitorum 2,3 (ED2,3) exhibited reciprocal pattern in relation to execution of static force between the index finger and thumb. However, after recovery from the C5 l-CST lesion, all the recorded muscles, including ADP and ED2,3, exhibited coactivation. This would reflect that the monkey tried to enhance the stiffness of the hand to compensate for the loss of force in general. Thus, even though the monkey apparently recovered the ability of fractionated digit movements, the strategy to control the muscle synergy changed. This may suggest that PNs by themselves have limitation in the capacity to control the finest movements of individual digits. We also recorded the LFP in the hand region of M1 and analyzed the coherence with the EMG activity. Before the lesion, coherence between the cortical LFP and EMG activity of distal hand muscle such as ADP could be observed with the peak at 17 Hz (β-band cortico-muscular coherence) (75). However, after the C5 l-CST lesion, the cortico-muscular coherence did not recover 3 months after the lesion, even when the ability of precision grip was almost completely recovered, which supports the suggestion in previous studies that the direct CM connection is required for the β-band cortico-muscular coherence (76, 77). At this stage, instead of the cortico-muscular coherence, prominent coherence between muscles spanning from the proximal to distal, which was not observed before lesion) appeared with the peak around 33 Hz (γ-band musculo-muscular coherence) (73). Thus, again here, motor strategy was switched after the l-CST lesion. These results suggest that even though the fractionated digit movements could apparently be recovered by the function of the PNs, the PNs cannot fully take over the function of the direct CM pathway [or the cortical inputs to the cervical enlargement (C6-Th1)].

## Conclusion

As reviewed above, the lesion of the corticofugal fibers causes different effects on the hand dexterity depending on at which level the region was made. Lesions made at the brainstem resulted in permanent impairment of hand dexterity, although the less fractionated power grip and reaching with more proximal muscles showed recovery. On the other hand, interestingly, the l-CST lesion made at the C5 resulted in fairy good recovery of hand dexterity. Capacity of recovery may reflect the available indirect pathways in each case. In case of lesion at the brainstem and the upper cervical spinal cord, the RuSNs and substantial part of RSNs should be available, which could control reaching with proximal muscles and less dexterous grasping (power grip), however, the capacity of these indirect pathways is not sufficient for recovery of the fractionated digit movements. In case of the lesion at the C5, the PNs in the mid-cervical segments are available, which can at least partly control the fractionated digit movements. But if the lesion was extended more ventrally and covered the axons of PNs and RSNs (hemisection), only the pathway descending in the contralateral spinal half could be available. These pathways can compensate for reaching and power grip, however, they are not sufficient for fully compensate for the dexterous hand movements. The ipsilateral motor cortex has been shown to contribute partly to the recovery of precision grip after the C5 l-CST lesion as revealed by local inactivation technique with muscimol (60), it may not be able to control the dexterous digit movements by itself. Based on these observations, it is likely that the potential of compensation for the hand dexterity varies among different subsets of neuronal populations that mediate cortical commands to motoneurons. Therefore, the appearance of motor deficits caused by the damage to the corticofugal fibers and expectation of recovery should be different depending on the rostrocaudal level of the lesion.

The existence and role of the indirect pathway from the motor cortex to hand and arm motoneurons, especially those relayed by the PNs has been debated (4, 8, 44, 78). The PNs were shown to control less dexterous movements such as reaching with proximal muscles in cats. In higher primates, the direct CM connection was developed and therefore the PNs which might have more divergent connection with distal motoneurons might not be beneficial for the control of fractionated digit movements (25). However, our recent studies showed that PNs in primates are involved in the control of dexterous digit movements with most distal muscles in the intact state (26, 27) and can be involved in the recovery of precision grip (as discussed above). Thus, along with the evolutional development of hand dexterity, the function of spinal circuits, especially those of the PN system, seems to have changed. Such evolutional aspect should be taken into account when we think of the hierarchy of the motor system, especially in the clinical context.

## Conflict of Interest Statement

The authors declare that the research was conducted in the absence of any commercial or financial relationships that could be construed as a potential conflict of interest.
